# Assessment of medicines use pattern using World Health Organization’s Prescribing, Patient Care and Health facility indicators in selected health facilities in eastern Ethiopia

**DOI:** 10.1186/s12913-016-1414-6

**Published:** 2016-04-23

**Authors:** Arebu I. Bilal, Ebrahim D. Osman, Anwar Mulugeta

**Affiliations:** Department of Pharmaceutics and Social Pharmacy, Addis Ababa University, College of Health Sciences, School of Pharmacy, P.O. Box 1176, Addis Ababa, Ethiopia; Department of Pharmacy, Jigjiga Health Sciences College, Jigjiga, Ethiopia; Department of Pharmacology, Addis Ababa University, College of Health Sciences, School of Medicine, Addis Ababa, Ethiopia

**Keywords:** Essential medicines list, Ethiopia, Health facility indicators, Patient care indicators, Prescribing indicators, World Health Organization

## Abstract

**Background:**

About one-third of the world’s population lack access to essential medicines and this is further compounded by inappropriate prescription, dispensing, sale and use of the available medicines. The objective of the study was to assess the patterns of medicine use among health facilities in eastern Ethiopia using World Health Organization’s Prescribing, Patient Care and Health facility indicators.

**Methods:**

A cross sectional study was carried out in eight randomly selected health centers and data were collected retrospectively as well as prospectively. Prescribing indicators were assessed retrospectively using 636 prescriptions selected by systematic random sampling technique among prescriptions filled between September 2013 and September 2014. Patient care indicators were assessed prospectively by interviewing 708 patients from the health facilities. Health facilities were assessed through observation. Data were entered and analyzed using Statistical Packages for Social Sciences version 20. *P*-value less than 0.05 at 95 % confidence interval considered for significance of relationships for associations in statistical tests.

**Results:**

The average number of medicines per prescription was 2.2 with standard deviation of 0.8. The proportion of medicines prescribed by generic name was 97 and 92 % of the prescribed medicines were included in List of Essential Medicines for Ethiopia, Prescriptions containing antibiotics and injections constituted (82.5 and 11.2 %) respectively. Of the total of 1426 medicines prescribed, 49.6 % were antibiotics, with amoxicillin (33.3 %) and co-trimoxazole (16.0 %) being the most commonly prescribed agents. The average consultation and dispensing times were 5.6 and 2.7 min, respectively. Among the medicines dispensed, 64.0 % were adequately labeled and the proportion of patients with adequate knowledge about medicines was 69 %.

**Conclusion:**

The prescribing and dispensing practices in the health facilities are fairly good and are not that far from the standard WHO requirements. However, there is a need to do more on some issues, including prescribing practice of antibiotics, average number of medicines per prescription, and patients’ dosage form knowledge.

**Electronic supplementary material:**

The online version of this article (doi:10.1186/s12913-016-1414-6) contains supplementary material, which is available to authorized users.

## Background

Rational use of medicines is observed when patients receive medications appropriate to their clinical needs, in doses that meet their own individual requirements, for an adequate period of time, and at the lowest price [1]. Despite this fact, one-third of the world population lacks access to essential medicine, more than 50 % of all medicines are used d inappropriately and 50 % of the patients have problem of compliance [2].

Irrational use of medicines is a common phenomenon in developing countries causing poor and costly services [3]. Practices like poly-pharmacy, use of wrong or ineffective medicines, underuse or incorrect use of effective medicines, use of combination products, which are often more costly and offer no advantage over single compound products, and overuse of antimicrobials and injections are the most common ones [4]. Reduction in quality of pharmacotherapy, wastage of resources, high treatment cost, high risk of adverse medicine reactions, and emergence of medicine resistance are negative consequences caused by irrational use of medicines [5]. Although there have been tremendous improvements in the pharmaceutical sector in the recent past, there is still a need to emphasize on the setting up of appropriate systems to monitor the rational use of medicine regularly [6].

Assessment of the pharmaceutical sector in 17 hospitals in Ethiopia conducted in 2014 showed that the average availability of key medicines in the stores was and dispensaries were 82.3 and 81.5 % respectively [7]. In addition, different researchers have reported the practice of irrational prescribing in Ethiopia. For instances, a study conducted in Hawassa Referral Hospital in the southern part of Ethiopia documented that the percentage of encounters with antibiotics and injections prescribed were 58.1 and 38.1 %, respectively [8]. Another study conducted in eight hospitals in southern Ethiopia revealed that there is a tremendous irrational prescribing practice in all the hospitals [9]. A retrospective study on prescription patterns of analgesics in 13 rural and regional hospitals reported that analgesics were prescribed for almost every patient indicating that, there was no a clear therapeutic guideline on prescribing analgesics [10].

Periodic assessment of medicines prescribing practices in a health facility will help to identify specific medicine use problems, sensitize practitioners on rational medicine prescription and provide policy makers with relevant information that could be useful in reviewing medicine-related policies [11]. The current study was, therefore, initiated in line with the aforementioned notions and used indicators stipulated by the World Health Organization (WHO).

## Methods

### Study area and period

The study was conducted in eight health centers found in Fafen Zone, which is one of the nine Zones in the Somali region found in the eastern part of Ethiopia. The zone is about 619 km east of Addis Ababa, the Capital of Ethiopia. The study was conducted from November to December 2014.

### Study design

A cross sectional study was carried out retrospectively and prospectively in eight randomly selected health centers in the zone. In assessing prescribing indicators, 636 prescriptions were selected from among those prescribed between September 2013 and September 2014. Status of the health centers in terms of patient care indicators, 708 patients who were getting services in the outpatient departments of the health centers were interviewed. For health facility indicators assessment observation, checking for the availability of key indicator medicines and essential medicines list/guidelines was conducted. All the three groups of indicators were assessed based on the WHO/International Networks for Rational Use of Medicines (INRUD) guidelines [2].

Data were collected by using pretested questionnaires for prospective study and WHO designed criteria based data collection formats for retrospective study. According to WHO/INRUD guide on how to assess medicine use at health institutions outpatient prescribing indicators includes average number of medicines per encounter, percentage of medicines prescribed by generic name, percentage of prescriptions with antibiotics, percentage of prescriptions with injections and percentage of prescribed medicines from essential medicines list (EML).

- The average number of medicines prescribed per encounter was calculated to measure the degree of poly pharmacy. Hence, it was calculated by dividing the total number of different medicine products prescribed to the number of encounters surveyed. Combination of medicines prescribed for one case was counted as one.
- Percentage of medicines prescribed by generic name was calculated to measure the tendency of prescribing based on the medicine’s generic name. It was calculated by dividing the number of medicines prescribed by generic name to the total number of medicines prescribed and multiplied by 100.
- Percentage of encounters with an antibiotic prescribed was calculated to measure the overall use of commonly overused and costly forms of medicine therapy. It was calculated by dividing the number of patient encounters prescribed with an antibiotic to the total number of encounters surveyed and multiplied by 100.
- Percentage of encounters with an injection prescribed was calculated to measure the overall level of commonly overused and costly forms of medicine therapy. It was calculated by dividing the number of patient encounters in which an injection was prescribed to the total number of encounters surveyed and multiplied by 100.
- Percentage of medicines prescribed from an EML was calculated to measure the degree to which practices are conformed to a national medicine policy as indicated in the national medicine list of Ethiopia. Percentage was calculated by dividing the number of products prescribed from essential medicines list to the total number of medicines prescribed and multiplied by 100.

Patient care indicators are average consultation time, average dispensing time, the percentage of medicines actually dispensed, the percentage of medicines adequately labeled and patient’s knowledge of correct dosage.

- Average consultation time was calculated by dividing the total time for a series of consultations to the number of consultations.
- Average dispensing time was calculated by dividing the total time for dispensing medicines to a series of patients by the number of encounters.
- Percentage of medicines actually dispensed was calculated by dividing the number of medicines actually dispensed at the health care facility to the total number of medicines prescribed and multiplied by 100.
- Percentage of medicines adequately labeled was calculated by dividing the number of medicine packages containing at least the medicine name, the strength and the frequency and length of time/day the medicine should be taken to the total number of medicine packages dispensed and multiplied by 100.
- Patient’s knowledge of correct dosage was calculated by dividing the number of patients who adequately reported the dosage schedules for all medicines to the total number of patients interviewed and multiplied by 100.

Facility indicators are EML availability, formulary availability, standard treatment guideline (STG) availability and key medicines availability. EML or Formulary or STG availability was determined during the time of the visit. Key medicines availability was calculated by dividing the number of specified products actually in stock to the total number of medicines on the checklist and multiplied by 100.

### Data collection and analysis

Two well-trained pharmacy personnel were recruited and deployed in each health centers. One of them was collecting prescribing indicators retrospectively by using prescriptions and prescription registration books, while the other was collecting the patient care indicators and the facility indicators prospectively. Specific types of data necessary to measure the prescribing indicators were recorded for each patient encounter and entered to the prescribing indicator form. According to the WHO guide on how to investigate medicine use in health facilities, at least 600 encounters should be included in a cross-sectional survey to describe the current prescribing practices, with a greater number if possible [12].

Statistical Packages for Social Sciences (SPSS) version 20 was employed for entry, and analysis of the quantitative data. The collected data were entered after being coded. In the statistical analysis frequencies, averages/means, standard deviations (SD) and percentages were obtained. In addition, one way analysis of variance (ANOVA) and Pearson’s chi-squared tests were done to check for associations among different variables. For statistical significance *p*-value less than 0.05 at 95 % confidence interval (CI) was considered.

### Ethical consideration

Ethical approval was obtained from Ethics Review Committee of the School of Pharmacy, Addis Ababa University (Ethical approval letter no ERB/SOP/01/10/2014). In addition, discussion about the aim and purpose of the survey was made with Fafen Zone Health Office and respective health institutions; permission was obtained from the respective health institutions to work in the setup. Individuals participating in the study were informed about the purpose, benefits and the potential risks of the study. Finally oral consent was obtained from each study participant before conducting the interview. Patient related data was confidential and was destructed after forming database.

## Results

In the assessment of WHO prescribing indicators all the 636 prescription encounters sampled all and all the 708 patient interviews were included in the final analysis while making a 100 % completion and response rates. Thus, the average number of medicines per prescription was 2.2 (SD = 0.8). Out of the total 1426 medicines prescribed, almost all (1385, 97 %) were prescribed by generic name while a similarly very high proportion (1311, 92 %) of medicines were from the EML of Ethiopia. Antibiotics were prescribed in more than four-fifths (525, 82.5 %) of the patient encounters while just above a tenth (71,11.2 %) of prescription encounters ended up with injections in them (Table 1).

**Table 1:** Summary of results obtained in eight selected health centers in Fafen Zone Eastern Ethiopia from September 2013 to September 2014. (*n* = 636 encounters)

Prescribing indicators assessed	Total drugs/encounters	Average/percent	Standard derived or ideal
Drugs per encounter	1426	2.2	(1.6–1.8)
Encounter with antibiotics	525	82.5 %	(20.0–26.8 %)
Encounters with injection	71	11.2 %	(13.4–24.1 %)
Drugs prescribed by generic name	1385	97 %	100 %
Drugs from essential drug list	1311	92 %	100 %

In terms of proportion out of the total number of medicines prescribed, antibiotics constituted almost half (708, 49.6 %). Among these, the most commonly prescribed were amoxicillin (236, 33.3 %), co-trimoxazole (114, 16.0 %) and ciprofloxacin (86, 12.0 %) (Table 2). On the other hand, the most commonly prescribed injections were diclofenac (26, 37.0 %), procaine penicillin fortified (17, 24.0 %) and gentamicin (13, 18.0 %) (Table 3).

**Table 2:** Most commonly prescribed antibiotics at the outpatient pharmacy of eight selected health centers in Fafen Zone Eastern Ethiopia from September 2013 to September 2014. (*n* = 636 encounters)

Commonly prescribed antibiotics	Frequency (Percentage (%))
Amoxicillin	236(33.3)
Metrondazole	71(10.0)
Cotrimoxazole	114(16.0)
Ciprofloxacillin	86(12.0)
Doxycycline	53(7.5)
Cloxacillin	22(3.0)
Ampicillin	30(4.0)
Norfloxacillin	17(2.4)
Procaine penicillin fortified	17(2.4)
Others^a^	62(8.8)

Others^a^: Benzyl penicillin, Gentamycin, Erythromycin, Claritomycin, Tetracycline, Chloroamphenicol and Ceftriaxone

**Table 3:** Most commonly prescribed injections at the outpatient pharmacy of eight selected health centers in Fafen Zone Eastern Ethiopia from September 2013 to September 2014. (*n* = 636 encounters)

Commonly prescribed injection	Frequency (Percentage (%))
Diclofenac	26(37.0)
Procaine penicillin fortified	17(24.0)
Gentamycin	13(18.0)
Benzyl penicillin	8(11.0)
Cimetidine	3(4.0)
Others^a^	4(6.0)

Others^a^: Ceftriaxone, Ampicillin, Chloroamphenicol and Hyoscine

Table 4 shows average consultation time, dispensing time, number of medicines prescribed, number of medicines dispensed, number of medicines adequately labeled, and the patients knowledge about the medicines dispensed to them, measured through prospective data collection involving 708 patients.

**Table 4:** Distribution of Patient care indicators of drug use at eight selected health centers in Fafen Zone Eastern Ethiopia, December 2014. (708 encounters)

Indicators	Name of health centers										
	Kebrebeyah local	Bombas	Kebrebeyah refugee	Awberae local	Lefeisae	Ayerdega	Jigjiga	Awberae refuge	Total	WHO Standards	*P*-Value
Average consultation time in minute (Range)	6.89 (2–10)	7.97 (3–15)	1.79 (1–4)	2.19 (1–3)	11.43 (5–19)	6.62 (3–10)	4.92 (2–9)	3.0 (1–8)	5.11 (1–19)	10	<0.001*
Average dispensing time in minute (Range)	2.0 (0.33–6)	3.7 (1–8)	0.46 (0.16–1)	1.7 (0.83–3)	5.0 (2–9)	2.2 (1–5)	5.0 (2–9)	1.6 (0.16–8)	2.6 (0.16–9)	>3	<0.001*
% of drugs actually dispensed	87.45	88.44	98.46	92.91	81.13	70.99	81.81	91.15	86.22	100 %	<0.001*
Average no of drugs dispensed per prescription (Range)	2.44 (0–4)	2.59 (1–5)	1.93 (1–4)	2.23 (1–4)	2.19 (1–3)	1.86 (0–4)	1.89 (1–4)	1.88 (1–4)	2.11 (0–5)		
% of drugs adequately labeled	70.77	65.22	45.96	60.66	85.53	55.45	75.87	51.55	60.56	100 %	<0.001*
Average number of drugs adequately labeled per prescription (Range)	2.41 (0–4)	2.60 (1–5)	1.96 (1–4)	2.23 (1–4)	2.2 (1–3)	1.87 (0–4)	1.92 (1–4)	1.90 (1–4)	2.11 (0–5)		
Patient Knowledge	82.5	36.8	84.0	72.0	74.1	85.0	43.0	73.3	69.8	100 %	<0.001**

*Result of one way ANOVA, **Pearsons Chi-square result

Among the eight health centers, the national EML was available only in six of the health centers at the time of the study. A shortlist of thirteen tracer medicines used to treat common health problems were made and the availability of these medicines in the pharmacy was evaluated (Table 5). Amoxicillin, mebendazole, paracetamol and gentamicin were found in all of the health centers, while oral rehydration salts (ORS), rifampicin, isoniazid, pyrazinamide and ethambutol (RHZE) combination and ferrous sulphate with folic acid were found only in one of the health centers. Tetracycline and medroxyprogesterone injection were found only in three health centers out of the eight health centers in the zone (Table 5).

**Table 5:** Availability of key drugs in eight selected health centers in Fafen Zone Eastern Ethiopia, December 2014

		Health facility name						
Tracer Drug	Kebrebeyah local	Bombas	Kebrebeyah refugee	Awberae local	Lefeisae	Ayerdega	Jigjiga	Awberae refuge
Amoxicillin	Yes	Yes	Yes	Yes	Yes	Yes	Yes	Yes
Oral Rehydration Salts	No	No	No	No	No	No	No	Yes
Arthemisin/Lumphantrine	Yes	No	Yes	No	Yes	Yes	Yes	No
Mebendazole Tablets	Yes	Yes	Yes	Yes	Yes	Yes	Yes	Yes
Tetracycline Eye Ointment	No	No	No	No	Yes	Yes	Yes	No
Paracetamol	Yes	Yes	Yes	Yes	Yes	Yes	Yes	Yes
Refampicine/Isoniazide/Pyrazinamide/Ethambutol	No	No	No	No	No	No	No	Yes
Medroxyprogesterone (depo) Injection	Yes	No	No	Yes	No	Yes	No	No
Ergometrine Maleate Tablets	Yes	No	Yes	No	Yes	No	Yes	No
Ferrous Salt plus Folic Acid	Yes	No	No	No	No	No	No	No
Pentavalent DPT-Hep-Hib Vaccine	Yes	No	Yes	No	Yes	No	Yes	Yes
Zinc	Yes	No	No	No	Yes	Yes	Yes	No
Gentamycin	Yes	Yes	Yes	Yes	Yes	Yes	Yes	Yes
% of availability of key drugs in stock	77 %	31 %	54 %	38 %	69 %	61 %	69 %	54 %

## Discussion

The average number of medicines per prescription in the present study was 2.2, which is higher than the ideal WHO standard (1.6–1.8) [12]. The values reported in this study are very much similar with some studies [13–16]. However, lower average numbers of medicines per encounter (1.2, 1.7, 1.8, and 1.9) were reported in different regions of Ethiopia [8, 17–20]. In addition, other studies outside Ethiopia, also reported a low number of medicines per encounter, for example, 1.4 (Sudan) [21] and 1.3 (Zimbabwe) [22]. Even though there are no adequate studies that identify the reasons for poly-pharmacy in the study area, it might be related to lack of adequate training of health professionals, variation in the health care delivery system, differences in socioeconomic profiles as well as morbidity and mortality characteristics of the population [8].

The percentage of medicines prescribed by generic name in this study was 97 %, which is close to the standard (100 %) [12]. The high level of generic prescription could probably attributed to the fact that the study was conducted in governmental health centers, where procurement of generic drugs is the prevailing practice. Similar findings have been reported in other studies conducted locally [8] as well as elsewhere [23]. Values which are significantly lower, ranging from 58–75 % than the present study have also been reported in different studies [16, 21, 22, 24].

The percentage of encounters in which antibiotics were prescribed in the study area was 82.5 %, which is high compared to the standard (20.0–26.8 %) [12]. A national baseline study on medicine use indicators in Ethiopia in 2003 also showed that, the percentage of encounters in which antibiotics were prescribed was 58.1 % [17]. Similarly, studies conducted in University Teaching and Referral Hospital and in four randomly selected health care facilities in southwest Ethiopia reported that, the percentage of encounters in which an antibiotics prescribed were 58 % and less than 30 % respectively [8, 24]. In the medicine use pattern study in 12 developing countries, the percentage of encounters with antibiotics prescribed were 63 % in Sudan, 56 % in Uganda, 48 % in Nigeria and 29 % in Zimbabwe [16, 21–23]. Besides, similar studies in Yemen, western China, and Nepal reported the percentage of encounters in which an antibiotic was prescribed as 66.2, 48.43, and 28.3 % respectively [3, 25, 26]. Over prescribing of antibiotic in this study might be due to mid level health professionals working in this area and small distribution of health professional to population ratio specially physician, health officers and pharmacist in the study area [27]. Over use of antibiotics, as it is the case in this study, may increase the chance of emergence of antimicrobial resistance [8].

In this study, the percentage of prescription with an injection encounter was 11.1 % which is lower than the standard (13.4–24.1 %) [12]. A higher percentage of encounter in which injections were prescribed at Hawassa University Teaching and Referral Hospital was 38.1 % [8]. In a prescription pattern study in 12 developing countries, the percentage of encounters in which injections were prescribed was high in Uganda (48 %) and Sudan (36 %) but very low in Zimbabwe (11 %) and in the acceptable range in Indonesia (17 %), Ecuador (17 %), and Mali (19 %) [16, 21–23]. Minimum use of injections is preferred as it reduces the risk of infection through parenteral route and cost incurred in therapy [3]. The lower prevalence of injection in this study might be the cultural barriers against injection based treatment in the study area.

The percentage of medicines prescribed from the essential medicine list for this study was 92 %, which is lower than the standard (100 %) [12]. This figure is lower than other studies in Ethiopia [8, 17]. In this study, the average consultation and dispensing time of facilities were 5.60 and 2.70 min respectively, which was similar with the study conducted in north west and south west Ethiopia where the average consultation times were 5.8 and 6.14 min respectively; and the average dispensing times were 1.9 and 1.28 min respectively [24, 28]. In fact, a shorter dispensing time (22.5 s) was reported on a study conducted in Jimma University Specialized Hospital [18]. Another study which was conducted in Niger reported a 5.75 min of consultation time which was similar to our finding but 3.25 min of average dispensing time [29]. However; another study in Jordan and Cambodia documented that 3.90 and 4.43 min of average consultation times and 28.80 s and 3.92 min of average dispensing times respectively [13, 30]. In this study, the average consultation time and dispensing time in some health centers were short. For instance in Kebrebeyah and Awberae Refugee Health Centers, the average consultation times were 1.79 and 3.0 min and the average dispensing times were 0.46 and 1.6 min respectively. Shorter consultation and dispensing time may lead to inadequate information about the medication being given to patients and patients had little chance to obtain information about their treatment. The potential reason for this variation can be due to differences in man power, patient overload, set up of dispensary area and ease of access for essential materials like medicines, medical equipment among health facilities.

The finding revealed that, on average 64 % of dispensed medicines were adequately labeled which is close to the study conducted in southwest Ethiopia, 70 % [24] and a study conducted in Islamic Republic of Iran, 60 % [31]. In other studies, all dispensed medicines were improperly labeled in Cambodia [31] and only1.4 % of prescriptions was adequately labeled in Nepal [3]. The study also showed that, 69.0 % of patients were able to repeat the correct dosage schedule of the medicine they had received which was relatively lower when compared to the other studies conducted in south west Ethiopia which were 79 and 72.80 % [19, 22], but higher than a study conducted in Cambodia [30].

### Limitations

The study used the WHO prescribing indicators, which are supposed to record exactly what is prescribed to patients, but not why. In order to explain why, studies in line with that have to be conducted.

## Conclusion

On the basis of the finding of this study, the prescribing practices for antibiotic and average number of medicines per prescription showed deviation from the standard recommended by WHO. There is a need to improve patients’ knowledge on dispensed medicines by increasing the dispensing time and improving the percentage of medicines adequately labeled. The availability of key medicines in the stock should be improved. Medicine use evaluation should be done for some of the antibiotics to check whether they were appropriately prescribed or not. On the other hand, use of injection, generic prescribing and prescribing from EML were not found to be a problem in this study. Baseline data gathered by this study can be used by researchers and policymakers to improve prescribing practices at the studied health centers.

## Ethics approval and consent to participate

This research received ethics approval from the Ethics Review Committee of the School of Pharmacy, Addis Ababa University (Ethical approval letter no ERB/SOP/01/10/2014).

## Availability of data and materials

The data supporting the conclusions of this article are available in the Additional files 1, 2 and 3.

## Additional files

Additional file 1: Patient care indicators data. (SAV 14 kb) Additional file 2: Prescribing care indicators data. (SAV 10 kb) Additional file 3: Health facility indicators data. (SAV 2 kb)

## Abbreviations

EML: essential medicines list
INRUD: International Network for the Rational Use of Medicines
ORS: oral rehydration salt
RHZE: rifampicin, isoniazid, pyrazinamide and ethambutol
SD: standard deviation
SPSS: Statistical Package for Social Sciences
STG: standard treatment guidelines
WHO: World Health Organization
